# Development and Application of a Dissolution-Transfer-Partitioning System (DTPS) for Biopharmaceutical Drug Characterization

**DOI:** 10.3390/pharmaceutics15041069

**Published:** 2023-03-26

**Authors:** Christian Jede, Laura J. Henze, Kirstin Meiners, Malte Bogdahn, Marcel Wedel, Valeria van Axel

**Affiliations:** 1 Global Analytical Development, Global CMC Development, Merck Healthcare KGaA, Frankfurter Strasse 250, 64293 Darmstadt, Germany; 2 Institute for Pharmacy and Food Chemistry, University of Wuerzburg, Am Hubland, 97074 Wuerzburg, Germany; 3 Global Drug Product Development, Global CMC Development, Merck Healthcare KGaA, Frankfurter Strasse 250, 64293 Darmstadt, Germany

**Keywords:** in vitro dissolution testing, in vitro dissolution methods, in vivo dissolution, oral drug absorption, oral bioavailability, gastrointestinal

## Abstract

A variety of in vitro dissolution and gastrointestinal transfer models have been developed aiming to predict drug supersaturation and precipitation. Further, biphasic, one-vessel in vitro systems are increasingly applied to simulate drug absorption in vitro. However, to date, there is a lack of combining the two approaches. Therefore, the first aim of this study was to develop a dissolution-transfer-partitioning system (DTPS) and, secondly, to assess its biopredictive power. In the DTPS, simulated gastric and intestinal dissolution vessels are connected via a peristaltic pump. An organic layer is added on top of the intestinal phase, serving as an absorptive compartment. The predictive power of the novel DTPS was assessed to a classical USP II transfer model using a BCS class II weak base with poor aqueous solubility, MSC-A. The classical USP II transfer model overestimated simulated intestinal drug precipitation, especially at higher doses. By applying the DTPS, a clearly improved estimation of drug supersaturation and precipitation and an accurate prediction of the in vivo dose linearity of MSC-A were observed. The DTPS provides a useful tool taking both dissolution and absorption into account. This advanced in vitro tool offers the advantage of streamlining the development process of challenging compounds.

## 1. Introduction

An increasing number of new chemical entities (NCEs) in pharmaceutical R&D belong to Biopharmaceutics Classification System (BCS) class II and IV [1]. For them, poor solubility in the intestinal fluids is a key parameter limiting oral drug absorption. Hence, the understanding of intraluminal mechanisms of BCS class II and IV compound absorption is crucial to forecast their in vivo behavior. To identify the underlying mechanism of poor exposure of these emerging drug candidates early in the development and to improve the compound’s developability, bio-predictive in vitro tools are key to investigating the drug dissolution, precipitation, and absorption behavior. To gain appropriate predictability with in vitro models, the models often aim to mimic physiological conditions of the human gastrointestinal (GI) tract. However, the employed standard in vitro models are often lacking bio-predictivity due to, e.g., the lack of an absorptive sink, GI transfer, or also formulation digestion.

The transit of the drug through the GI tract is a key parameter impacting intestinal drug absorption. When considering the dissolution of a drug in vivo, it is crucial to assess the dynamic dissolution process, including potential drug supersaturation, precipitation, and re-dissolution in luminal contents. Further, the potential re-dissolution of a precipitated drug is often underestimated during the in vitro characterization of new drugs and their formulations, whereas the role of in vivo re-dissolution is not yet fully understood [2]. Therefore, assessing supersaturation, precipitation, and re-dissolution is critical, especially in cases where the drug is a weak base with low aqueous solubility at neutral pH [2]. While a high solubility may be observed for weakly basic drugs in the acidic environment of the stomach, the solubility of such drugs is limited in the more neutral small intestine. Thus, supersaturation may occur after transfer from the more acidic stomach to the small intestine. While supersaturation can drive absorption and improve bioavailability by increased absorption, the supersaturated states are thermodynamically unstable, and the degree of supersaturation is the driving force for precipitation which may hamper absorption depending on the drug of interest [3]. Therefore, weakly basic drugs also carry the risk of solubility limited absorption and hence bioavailability due to precipitation on the transit through the GI tract. Consequently, the usage of in vitro technologies reliably assessing the potential luminal supersaturation/precipitation of new drug molecules to predict oral drug absorption in humans has increasingly gained attention over the last decade. However, as stated in a recent UNGAP review, ‘the ability to accurately predict solid form, particle size and re-dissolution for precipitated weak bases after gastric transfer remains beyond the current capabilities of the typical biopharmaceutics toolkit’ [2].

In vitro transfer models are well described in the literature, representing a two-compartmental dissolution model, mimicking the transfer of drug from the simulated stomach compartment to the simulated small intestine compartment, with the aim to replicate the crucial pH transit at this important junction of the GI tract [4,5,6,7,8]. The test setup is either a small-scale setup (1:10 scale-down), with the advantage of using only a small amount of drug, which is especially utilized in early phases of drug development, or full-scale using the standard USP II vessel of 1000 mL [4,5,6,7,8]. A key advantage of the two-compartmental models is the possibility to mimic the gradual transfer from one vessel to another, i.e., simulating relevant and variable GI transfer rates. In the case of ‘one-pot’ methods, a rapid shift from gastric to intestinal conditions is induced, which may lead to an overestimation of drug precipitation depending on the drug investigated [9]. However, both ‘standard’ two- and one-compartmental in vitro setups have in common that absorption is not taken into consideration. The lack of an absorption compartment is often described as one major limitation of these systems. As a result, many of the in vitro transfer models overestimate the degree of precipitation in the luminal compartment [10].

As a result of this limitation, various concepts have been explored to incorporate the absorption of the drug in the small intestine into in vitro models. One example is the use of an organic layer on top of the aqueous layer. In these models, the drug initially dissolves in the aqueous phase and partitions from the aqueous (simulated intestinal phase) to the organic layer mimicking an absorptive sink. Drug partitioning into the absorptive sink reduces the apparent drug concentration in the aqueous phase, thus, influencing the driving force for drug precipitation in the luminal fluids significantly. These biphasic dissolution models described in the literature have been developed with the purpose of simultaneously characterizing the dissolution and absorption process using predominantly a one-vessel setup [11,12,13,14,15]. In the latest years, combined biphasic USP apparatus II and IV settings gained attention in evaluating bio-enabling formulation strategies, with a promising outlook in improving prototype formulation selection of weak acid drug candidates [16,17]. However, in these studies, a rather small volume of 12 mL simulating the acidic stomach environment has been employed. Further, the system operated in a closed-loop configuration, thus, not including physiologically relevant emptying kinetics.

Combining the biphasic in vitro approach with the advantages of a USP II-based transfer model using 250 mL SGF transferred into ≥250 mL FaSSIF could simulate the drug removal (simulated absorption) during/after drug transfer and pH shift along the GI tract more closely to human intestinal conditions. Consequently, this could lead to an improved understanding of the in vivo performance of weakly basic drugs. However, to date, there is a lack of such an in vitro tool, which may be essential to improve the interpretation and forecast of in vivo results of weakly basic and poorly water-soluble drugs.

This study combines the in vitro USP II-based transfer model with an additional organic phase on top of the medium simulating fasted state conditions in human intestinal regions. This combined approach aims to reduce the risk of overestimating drug precipitation and supersaturation. Moreover, potential re-dissolution events should be more accurately captured. The presented work focused on the development of the dissolution-transfer-partitioning system (DTPS), which consists of a USP II paddle apparatus, a peristaltic pump, and a novel 3D-printed dynamic biphasic paddle for stirring both the aqueous and the organic phase in a height-adjustable fashion. For this purpose, two important parameters on drug supersaturation and precipitation were assessed, namely volume ratio (VR) between the aqueous and the organic phase, as well as the rate of first-order simulated GI transit. A weakly basic BCS class II drug MSC-A (Merck Healthcare KGaA development compound) was chosen as the model compound, which exhibits poor aqueous solubility at neutral pH. A further aim of the present study was the application of the DTPS to improve predictions of in vivo dose linearity of MSC-A via a comparison of obtained in vitro drug concentrations to in vivo PK data from a Ph1 study. For this evaluation, an oral solution was used at 75, 200, and 500 mg in the DTPS. Further, the performance of the DTPS was compared to the available USP II-based in vitro transfer model to judge the utilization of the model for the evaluated drug substance. Consequently, the work provides new insights into the use of an innovative bio-predictive in vitro tool using a model drug with poor biopharmaceutical properties that are representative of biopharmaceutical challenges in pharmaceutical R&D. Further, the model described herein offers great opportunities to better characterize enabling formulations that aim to induce a supersaturated and thermodynamic unstable state [18].

## 2. Materials and Methods

MSC-A (Table 1; purity ≥ 99%), a weakly basic Merck Healthcare KGaA development compound (parent form), was synthesized internally (Merck Healthcare KGaA, Darmstadt, Germany). Sodium chloride, sodium hydroxide, hydrochloric acid 37%, sodium dihydrogen phosphate, acetonitrile of HPLC gradient grade, and ammonia solution 25% were obtained from Merck KGaA (Darmstadt, Germany). Whatman PTFE syringe filters 0.45 µm were purchased from GE Healthcare UK Limited (Little Chalfont, UK). SIF powder was purchased from biorelevant.com (London, UK). All other chemicals were of analytical grade. Relevant physicochemical parameters of the MSC-A are given in Table 1.

### 2.1. Preparation of Oral Solutions of MSC-A for In Vitro Experiments and In Vivo Clinical Trials

For the preparation of the oral solutions (OS), 2000 mL of a 0.1 M phosphate buffer pH 2.1 was prepared, including 13.8 g NaH_2_PO_4,_ 200 g sucrose, 11.62 g of phosphoric acid (85%), and water for injection.

Finally, 2.5 g MSC-A drug substance and 5 g of orange flavor were weighed and transferred quantitatively into a 1000 mL volumetric flask and filled up to 1000 mL calibration mark with phosphate buffer, yielding a solution with a concentration of 2.5 mg/mL of MSC-A.

### 2.2. In Vivo Clinical Trials

The pharmacokinetics (PK) of MSC-A were assessed in a randomized First-in-human (FIH) clinical trial using single ascending doses (SAD) of 75, 200, and 500 mg administered as OS (see Section 2.1) to healthy male and female subjects under fasting conditions. In each of the studies, administration of the OS was performed together with a glass of water. As described in Table 2, the total administration volume was 240 mL for each dose level. Clinical trials were commenced after the provision of a written favorable opinion or approval from the Independent Ethics Committee (IEC) or Institutional Review Board (IRB). All clinical trials were performed in accordance with the protocol and with the ethical principles that have their origin in the Declaration of Helsinki, as well as with the International Conference on Harmonization (ICH) Guidelines for Good Clinical Practice (GCP) (ICH Topic E6, 1996) and applicable regulatory requirements.

### 2.3. In Vitro Experiments

#### 2.3.1. Dissolution Media

SGFsp (Simulated Gastric Fluid sine pepsin) was adjusted to pH 2 rather than pH 1.2 in order to reduce a substantial decrease in pH during the in vitro transfer of SGF into FaSSIF (final pH of 6.2 for experiments using 250 mL of SGF and FaSSIF; final pH of 6.3 for experiments using 250 mL of SGF and 350 mL of FaSSIF). The biorelevant medium FaSSIF (Fasted State Simulated Intestinal Fluid) pH 6.5 was prepared using a double-concentrated phosphate buffer to maintain the pH of the medium at a constant and physiologically relevant level during transfer experiments as described in a previous publication [19]. This resulted in 56.8 mM NaH_2_PO_4_ and a corresponding buffer capacity of 20 mM. For the development of the DTPS, 1-decanol was used as an organic simulated absorptive sink (see Section 2.3.4).

#### 2.3.2. Solubility Tests

Solubility of MSC-A in both SGFsp pH 2.0 and FaSSIF pH 6.5 (Table 1) has been reported previously [6]. Solubility in 1-decanol was assessed using the shake-flask method at 37 ± 0.5 °C by adding an excess amount of drug substance to preheated 1-decanol. After 24 h, samples were withdrawn and filtered through a 0.45 µm PTFE filter with subsequent dilution of the supernatant with 1-decanol for HPLC analytics.

#### 2.3.3. In Vitro Transfer Model

A USP II-based in vitro transfer model was employed for testing the dose-dependent supersaturation potential of MSC-A. In general, this in vitro model comprises three mini (500 mL) and three standard (1000 mL) dissolution vessels. The rotation speed was set to 75 rpm and the temperature to 37 ± 0.5 °C throughout the entire experiment. API (or API within a dosage form) was pre-incubated/pre-dissolved for 5 min in a simulated stomach (donor, mini vessel) containing approximately 250 mL SGF pH 2.0 and, subsequently, transferred into a simulated small intestine (acceptor, standard vessel), containing 350 mL FaSSIF pH 6.5, using an ERWEKA^®^ DT80 USP II dissolution tester (Erweka^®^ GmbH, Heusenstamm, Germany). The two vessels were connected via a programmable peristaltic pump (ISMATEC^®^ MCP Process IP65, IDEX Health & Science, Wertheim, Germany) to allow a physiologically relevant first-order simulated gastric emptying with a half-life of 5 min (total transfer duration of 25 min). In the case of testing disintegrating tablet formulations, the dosage forms were not completely dissolved at the time of starting the simulated GI transfer experiment. In the case of testing OS, the API was already in a dissolved state at this time point. The apparent concentration of MSC-A was determined at predefined time points by taking samples from the simulated intestinal phase compartment. Therefore, 3 mL were withdrawn and filtered as described in Section 2.3.2. The first approximately 2 mL were replaced into the vessel during filtration, whereas 300 µL of the remaining filtrate was diluted 1:1 with acetonitrile/water (50:50) before HPLC analytics to prevent drug precipitation. For investigating the three different doses tested in the clinical trials (see Section 2.2), the same conditions as in the clinics were mimicked, i.e., 210 mL SGF pH 2.0 in addition to 30 mL of OS in case of 75 mg, etc. All experiments were performed in triplicate in a parallel design to reduce the variability of data.

#### 2.3.4. In Vitro Dissolution-Transfer-Partitioning System (DTPS)

**Figure 1:**
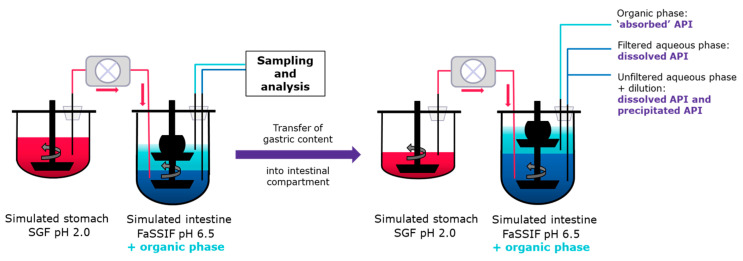
Schematic representation of the dissolution-transfer-partitioning system (DTPS), including the 3D printed dynamic biphasic dissolution paddle device and sampling information.

For the development of the DTPS, the USP II-based in vitro transfer model was used as a basis. As for the transfer model, the rotation speed was set to 75 rpm and the temperature to 37 ± 0.5 °C throughout the entire experiment. The major difference to the in vitro transfer model is the addition of an organic phase, in this study 1-decanol, on top of FaSSIF that simulates the small intestinal region. The addition of 1-decanol allows mimicking drug removal, i.e., simulated drug absorption through the gut wall. As described by Denninger et al., 1-decanol exhibits a lower solubility in water compared to commonly used 1-octanol. Further, the bad odor of 1-octanol reduces user-friendliness [20]. To allow for constant mixing within the organic phase, a second dissolution paddle was added (Figure 1). This paddle was manufactured by 3D printing using fused deposition modeling (FDM, see Section 2.3.5). In contrast to the in vitro transfer model experiments, three samples from the biphasic acceptor compartment were analyzed at each time point. The first sample was taken from the aqueous phase using glass syringes, i.e., FaSSIF. Subsequently, this aqueous sample was divided into two sub-samples. The first sub-sample was filtered and diluted as described above to account for the dissolved amount. In contrast, the second sub-sample was diluted until complete dissolution with organic solvent without any filtration to account for the total drug amount in the aqueous phase, undissolved/precipitated and dissolved drug. The second sample was taken from the organic phase (using glass syringes) to determine the drug concentration and partitioning into 1-decanol (see also Figure 1). 

The surface of a dissolution vessel is quite limited, approximately 78 cm^2^. The aqueous volume (total volume after GI transfer) is 500–600 mL in the present study. Consequently, this corresponds to area-to-volume ratios of 0.16 cm^−1^ and 0.13 cm^−1^ which is in line with previously reported values used for in vitro biphasic dissolution tests [21]. At the same time, in vivo, the human absorption surface area in the intestine is reported to be ‘in the order of half of a badminton court’ [22]. As described by Mudie et al., the physiological area-to-volume ratio for humans can be estimated to be between 1.9 cm^−1^ and 2.3 cm^−1,^ considering the compression of the intestine [23]. Thus, increasing the surface area of the dissolution equipment would be required, as described by researchers in the past [24]. However, the aim of this study was to develop a USP II-based advanced in vitro dissolution model that is based on an established and easy-to-implement transfer model setup. Since the geometry of a compendial dissolution vessel is fixed and does not allow for modifications, the impact of aqueous to organic solvent VR on MSC-A supersaturation/precipitation and partitioning was assessed using 75 mg immediate release (IR) tablets of MSC-A. For this purpose, a VR of 0.4 was compared to a VR of 0.8. VR 0.4 represents 250 mL SGF, transferred into 350 mL FaSSIF with a 1-decanol layer of 250 mL (600 mL aqueous phase: 250 mL organic phase), whereas VR 0.8 represents 250 mL SGF, transferred into 250 mL FaSSIF with a 1-decanol layer of 400 mL (500 mL aqueous phase: 400 mL organic phase). Further, given the importance of physiological variability, the impact of the rate of simulated GI transit was assessed as well. As described by Grimm et al., gastric emptying was observed to show high variability between as well as within subjects, even under fasted state conditions in humans [25]. Thus, two physiologically relevant first-order GI transit rates, 5 min vs. 9 min (t_1/2_), were applied in the first experiments using 75 mg (IR) tablets of MSC-A (total transfer duration of 25 min and 45 min, respectively). For investigating the three different doses tested in the clinical trials (see Section 2.2), the same conditions as in the clinics were mimicked, i.e., 210 mL SGF pH 2.0 in addition to 30 mL of OS in case of 75 mg, etc. All experiments were performed in triplicate in a parallel design to reduce the variability of data.

#### 2.3.5. 3D Printing of Dynamic Biphasic Paddle Device

**Figure 2:**
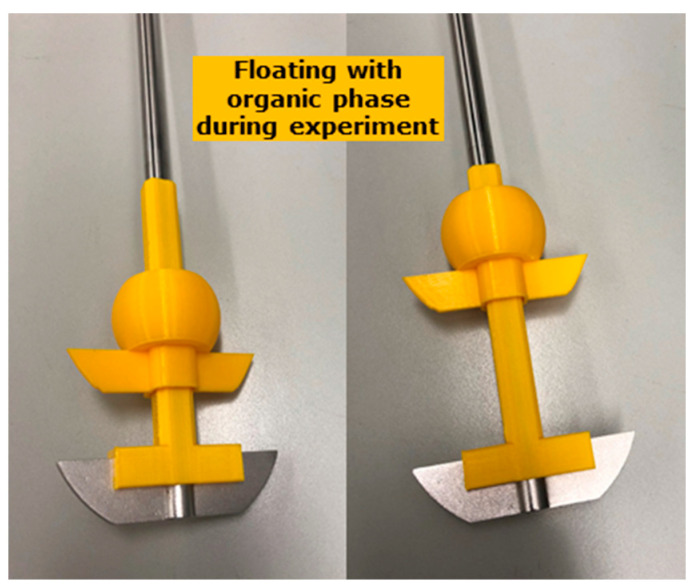
Representation of the 3D printed dynamic biphasic dissolution paddle device.

The floating paddle was designed to automatically adjust the position of the upper paddle following the fluid level during the transfer experiment. The floating paddle comprises an orifice with a square-shaped cross-sectional area. Furthermore, a sleeve was designed to be attached to the original shaft of the paddle without additional modifications or tools (Figure 2). After assembly, the shaft constrains the possible motion of the floating paddle in x- and y-direction synchronizing the rotational movement to the original paddle located at the bottom of the vessel. Motion in the z-direction, along the shaft, on the other hand, is not constrained, allowing upward and downward motion of the floating paddle. The buoyancy of the floating part was adjusted empirically so that the upper edge of the paddle was located 1 cm below the level of fluid. In the case of varying organic fluid volumes, the paddle position towards the aqueous/organic interface is different. Therefore, the distance between the blade and buoyancy body was adjusted empirically to allow a comparable position when using different organic fluid volumes. Thus, hydrodynamic differences should be reduced.

Solvent compatibility was tested by immersion of small pieces of material in the respective dissolution media. No relevant changes in the material properties were observed. Long term stability of the material in different dissolution media was not investigated in the scope of this study. To exclude drug partitioning into polylactic acid (PLA), recovery of MSC-A has been determined after all DTPS experiments. For this purpose, the gastric compartment and tubes were rinsed with 200 mL 0.1 N HCl after the experiment, followed by concentration determination. The sum of concentrations determined in all phases, i.e., total aqueous amount, organic, and aqueous after rinsing the setup, revealed a high recovery of ≥95%.

The two parts were manufactured using the 3D printing technique FDM. The parts were designed with Autodesk Fusion 360 and exported to stereolithography format (*.stl). These files were imported into Slic3r Prusa Edition (1.41.2 + win64) and sliced into gcodes using the settings as described in Table 3.

### 2.4. Quantitative Analysis of Samples

#### 2.4.1. In Vivo

For PK assessments, venous blood samples (2 mL) for determination of MSC-A concentration in plasma were collected per the schedule of assessments, including blood sampling after 0.25, 0.5, 1, 1.5, 2, 2.5, 3, 4, 5, 6, 8, 12 h. For the PK analysis, quantitative analysis of plasma concentrations of MSC-A was conducted at Q2 Solutions in Ithaca, New York, NY, USA, using a validated ultra-performance liquid chromatography-tandem mass spectrometry (UPLC-MS/MS) method (see Supplementary Materials). The calibration range was from 0.1 to 100 ng/mL. Assay precision based on the measurement of quality control samples was better than 4.6%, and accuracy was better than 4%.

#### 2.4.2. In Vitro

HPLC analytical finish was used to determine the dissolved/supersaturated, un-dissolved/precipitated, as well as the partitioned amount of MSC-A during in vitro transfer model and in vitro DTPS experiments. Therefore, an Agilent 1260 Infinity series HPLC system (Agilent Technologies, Santa Clara, CA, USA) was employed. The HPLC system consisted of a Binary Pump, an Agilent Autosampler, a Thermostat, a Thermostated Column Compartment, and an Agilent Diode Array Detector VL. The Agilent OpenLAB software (Agilent Technologies, Santa Clara, CA, USA) was employed. The method parameters used for drug quantification are summarized in the Supplementary Materials. LOD and LOQ determinations yielded appropriate results. Calibration curves were linear, with an R2 of 0.9999.

### 2.5. Data Analysis and Statistics

As described in Section 2.3.4, both filtered and non-filtered samples were analyzed to account for the dissolved amount as well as the total amount in the aqueous simulated intestinal compartment. The undissolved amount, the precipitated mass (mg) in the case of experiments using OS, was calculated as described by Equation (1). To account for the precipitated amount with respect to the applied dose, i.e., 75, 200, or 500 mg in OS, the cumulative transfer (theoretically transferred mass/mg) during the simulated GI transfer experiments was calculated according to Equations (2) and (3), with V0 representing the gastric volume at t = 0 and Vn representing the remaining gastric volume calculated for each time point following a first order emptying kinetic with the respective half-time. Subsequently, the precipitated amount (%) was calculated using the precipitated mass as a function of the theoretically transferred mass (mg) at the respective sampling time point, see Equation (4). In the case of using tablet formulations, the precipitated mass/amount (Equations (1) and (4)) must be considered as solid mass/amount consisting of undissolved and precipitated API).

(1) precipitated mass (mg)=total amount (mg)−dissolved amount (mg)

(2) $$transferred\;volume\;\left(mL\right)=Vt=V0-(\sum \;V_{n})$$

(3) $$cumulative\;transfer\;\left(mg\right)=concentration\;donor\;\left(\frac{mg}{mL}\right)\times \mathrm{transferred}\;\mathrm{volume}(mL)$$

(4) $$precipitated\;amount\;\left(\%\;of\;dose\right)=\frac{precipitated\;mass\;\left(mg\right)}{cumulative\;transfer\;\left(mg\right)}\;\times \;100\;$$

The area under the concentration-time curve (AUC) of the explored time frame was calculated based on the concentration-time profiles obtained from the in vitro transfer experiments.

To investigate the statistical difference between the studied factors (VR and transfer rate), a two-tailed *t*-test was applied (SigmaPlot version 14, Systat Software, Erkrath, Germany). A Mann-Whitney U test was applied if the normality or equal variance test, conducted prior to a *t*-test, was not passed. Ordinary least squares regression analysis assuming a linear dependence of y = mx of the in vivo and in vitro data, respectively, was performed using the statsmodels library (v.0.13.2) in Python 3.8.8. Significance was stated when the calculated *p*-value was found to be <0.05.

## 3. Results

### 3.1. Solubility Tests

MSC-A represents a weakly basic and strongly pH-dependent drug (as reported in Table 1). Hence, solubility in SGF at pH 2.0 is substantially higher compared to solubility in FaSSIF at pH 6.5. In line with the high lipophilicity of MSC-A (logP~3), the solubility in 1-decanol was determined to be 3.8 mg/mL using the shake flask method at 37 ± 0.5 °C.

### 3.2. In Vitro Dissolution-Transfer-Partitioning System (DTPS) Experiments

#### 3.2.1. Development of the DTPS

**Figure 3:**
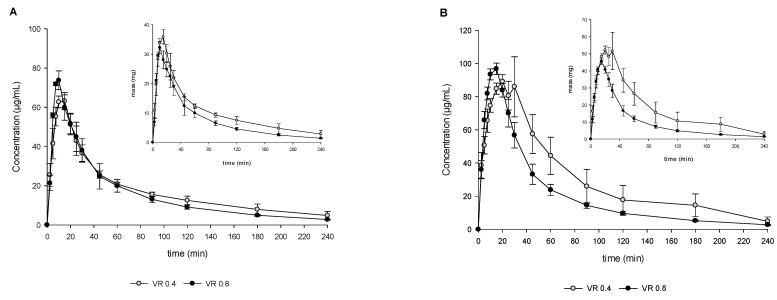

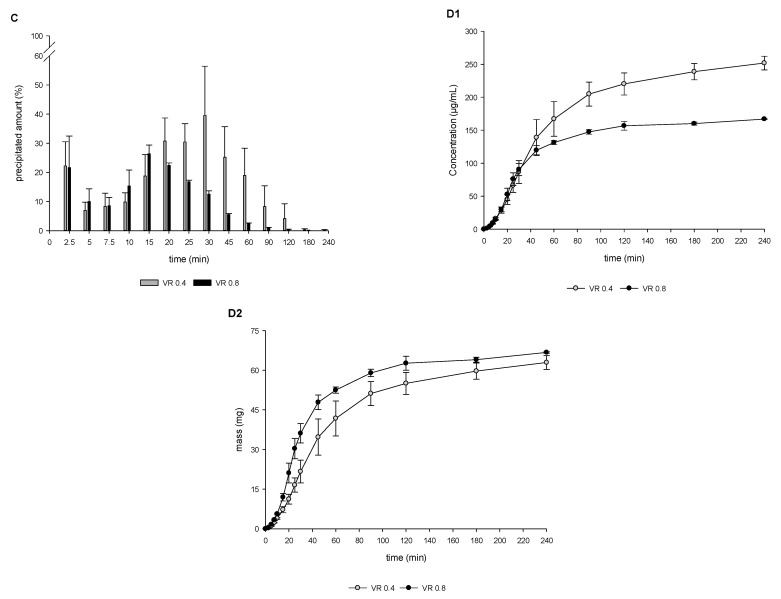
Drug concentration profiles (dissolved, (**A**)), total amount profiles (dissolved and undissolved/precipitate, (**B**)), and undissolved/precipitated amount (**C**) obtained from the aqueous filtered and un-filtered phase; partitioning profiles ((**D1**) concentration/time vs. (**D2**) mass/time) obtained from the organic phase during in vitro DTPS experiments using VR of 0.4 vs. 0.8 and 75 mg IR tablet formulation. The precipitated mass/amount (Equations (1) and (4)) must be considered as solid mass/amount consisting of undissolved and precipitated API. Means ± SD, *n* = 3.

For the development of the DTPS, two important parameters were assessed. The first principle was the VR between the aqueous and organic phase in the acceptor compartment simulating the intestine. Figure 3 summarizes the investigations in the DTPS using the two VR values (0.4 and 0.8) in the acceptor compartment. As depicted in Figure 3A, the amount of dissolved drug in the aqueous phase was similar for both VR and resulted in similar concentration over time profiles. In contrast, pronounced differences could be detected when comparing the total amount, i.e., precipitated and dissolved API, in the aqueous acceptor phase (Figure 3B). After 20 min of transfer, the precipitated amount started to differ significantly between the two VRs resulting in a peak of up to approximately 40% of the precipitated drug in the case of a VR of 0.4, while only approximately 10% of the drug precipitated in the case of a VR of 0.8 at 30 min (Figure 3C). As illustrated in Figure 3D1, major differences could be observed during later partitioning time points caused by the different VRs between the aqueous and organic phases. Therefore, volume differences between VR 0.4 and VR 0.8 were taken into account and normalized when calculating the transferred mass of API. Figure 3D2 shows that a faster mass transport/ partitioning into the organic layer was achieved in the case of a VR of 0.8 compared to 0.4 (statistically significant difference between VR 0.4 and 0.8 in the time frame between 5 min and 60 min, *p* < 0.05). For further experiments, VR 0.8 was selected since it is more in accordance with the observed in vivo data, i.e., lower precipitation in vitro using VR 0.8, and in vivo precipitation not impacting drug absorption as indicated by high dose linearity (see Section 4).

**Figure 4:**
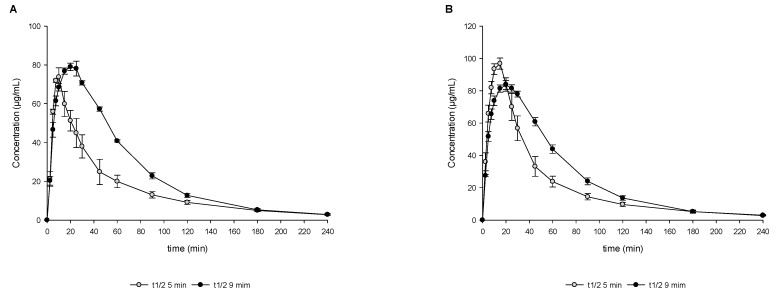

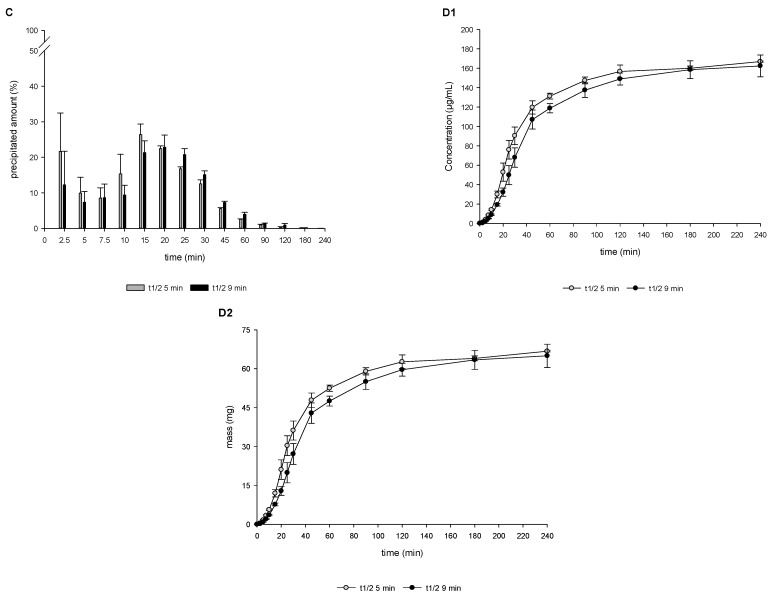
Drug concentration profiles (dissolved, (**A**)), total amount profiles (dissolved and undissolved/precipitate, (**B**)), and undissolved/precipitated amount (**C**) obtained from the aqueous filtered and un-filtered phase; partitioning profiles ((**D1**) concentration/time vs. (**D2**) mass/time) obtained from the organic phase during in vitro DTPS experiments using VR of 0.8 at simulated gastric emptying rates of 5 min half-time vs. 9 min half-time and 75 mg IR tablet formulation. The precipitated mass/amount (Equations (1) and (4)) must be considered as solid mass/amount consisting of undissolved and precipitated API. Means ± SD, *n* =3.

The second parameter investigated for the development of the DTPS was the transit rate. Figure 4 summarizes the investigations in the DTPS using two different physiologically relevant first-order GI transit rates, t_1/2_ 5 min vs. 9 min. As depicted in Figure 4A,D1,D2 a higher amount of dissolved drug could be observed in the case of t_1/2_ 9 min with an associated slower partitioning into 1-decanol. In contrast, the precipitated amount was found to be comparable between the two conditions, with the only marked difference expressed by a higher precipitated amount at the first sampling time points using t_1/2_ 5 min (Figure 4C). Partitioning into 1-decanol was found to be less impacted compared to the experiments using different VRs with a faster partitioning using t_1/2_ 5 min (statistically significant difference between VR 0.4 and 0.8 in the time frame between 5 min and 20 min, *p* < 0.05).

#### 3.2.2. Application of the DTPS to Investigate Dose Proportionality

**Figure 5:**
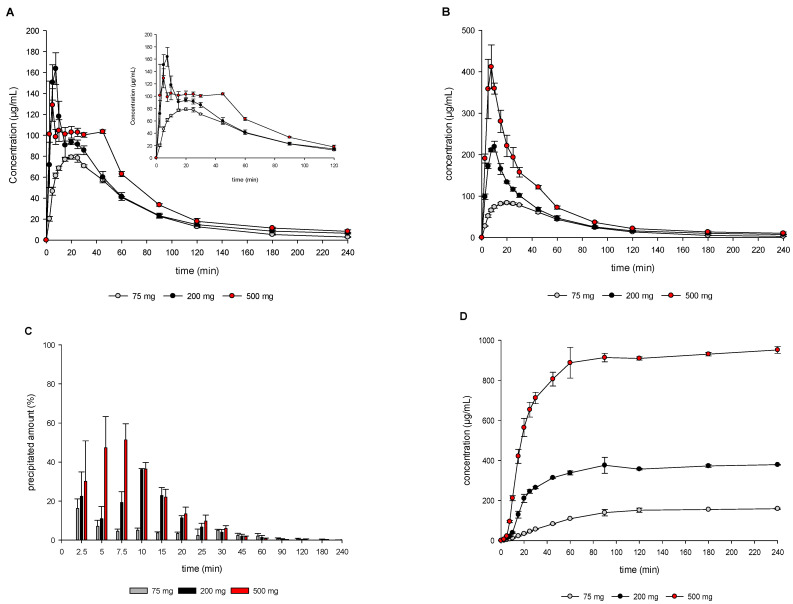
Drug concentration profiles (dissolved, (**A**)), total amount profiles (dissolved and precipitate, (**B**)), and precipitated amount (**C**) obtained from the aqueous filtered and un-filtered phase; partitioning profiles (**D**) obtained from the organic phase during in vitro DTPS experiments using 75, 200, and 500 mg MSC-A as OS; VR 0.8 and t_1/2_ 9 min. Means ± SD, *n* = 3.

Figure 5 shows the results of the in vitro dose escalation study (75 mg, 200 mg, and 500 mg OS) of MSC-A in the previously developed DTPS (at 75 mg dose in tablets). MSC-A supersaturation was found to be dose-dependent, i.e., higher supersaturation levels at higher doses. Further, a remarkable difference in drug precipitation was observed when using the DTPS. Interestingly, experiments using 500 mg resulted in a lower initial dissolved amount associated with a higher precipitated amount and faster drug partitioning compared to 200 mg. At higher dose levels of 200 mg and 500 mg, precipitation followed a two-step process with a plateau phase in between. In both cases, initial supersaturation (6.5-fold and 8-fold) resulted in precipitation which was followed by a plateau phase with constant drug concentrations at a supersaturation ratio of approximately 5-fold for both the 200 mg and 500 mg dose. Subsequently, a second drug precipitation event occurred, and drug concentration approximated to equilibrium concentration. As indicated by Figure 5B, the profiles of dissolved and undissolved (precipitated) drug were dose-dependent, with increasing precipitation levels at increasing doses. Consequently, Figure 5C shows the highest precipitated amount (%) for the experiment using 500 mg of MSC-A. Interestingly, for all dose levels, the precipitated amount (%) decreased again over the duration of the experiment (specifically after 2.5 min for 75 mg, after 7.5 min for 200 mg, and after 10 min for 500 mg), indicating drug re-dissolution. Drug partitioning into 1-decanol was observed to be significantly faster and higher when increasing the dose (Figure 5D), correlating with the degree of supersaturation.

### 3.3. In Vitro Transfer Model Experiments

**Figure 6:**
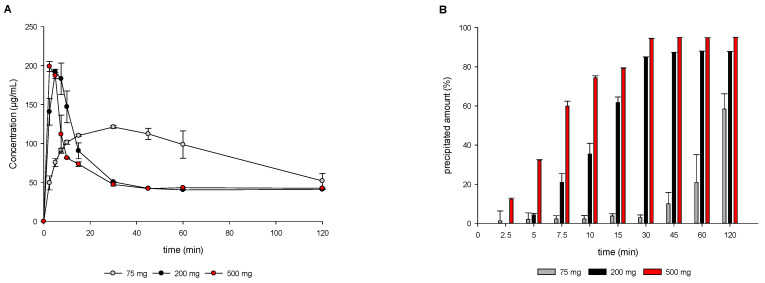
Aqueous phase drug concentration profiles (**A**) and precipitated amount (**B**) obtained from in vitro transfer model experiments using 75, 200, and 500 mg MSC-A as OS. Means ± SD, *n* = 3.

As illustrated in Figure 6A, the supersaturation potential showed a pronounced difference between 75 mg, 200 mg, and 500 mg OS as already observed using the DTPS. In the case of 75 mg OS, an approximately 5-fold supersaturation, compared to the thermodynamic solubility of MSC-A in FaSSIF pH 6.5, was observed. In contrast, analyzing the higher doses of 200 mg and 500 mg OS, supersaturation was observed to show substantially higher values up to approximately 10-fold supersaturation. Interestingly, on the one hand, the supersaturation potential significantly increased when increasing the dose from 75 mg to 200 mg, while the switch from 200 mg to 500 mg dose resulted in comparable supersaturation levels. In line with general supersaturation and precipitation theory, the precipitation rate was much faster after achieving higher supersaturation values compared to lower supersaturation values [3]. In contrast to the results observed using the DTPS, drug precipitation did not follow a two-step precipitation process. Instead, a zero order-like precipitation for the 75 mg dose and a first order-like precipitation for the higher doses of 200 mg and 500 mg were observed.

Figure 6B shows the precipitated amount of drug in the intestinal compartment in %, which was calculated based on the theoretically transferred drug amount at every sampling time point (see also Section 2.5). For the 75 mg OS dose, the precipitated amount was ≤20% until 60 min. Pronounced precipitation of the 75 mg dose occurred from the sampling time point of 60 min to 120 min. In contrast, for the higher doses of 200 mg and 500 mg OS, the overall precipitated fraction was found to be much higher, with >80% of the drug precipitated after 30 min.

## 4. Discussion

Using FDM, it was possible to manufacture a custom-made height-adjustable dynamic second dissolution paddle that can be optimized for different experimental settings, such as different organic fluid volumes. As a part of the development of the new system, attention was paid to the potential transfer of solid particles into the 1-decanol phase. This unwanted transfer could be excluded via visual observation, especially using tablets that contain non-dissolving excipients. The absence of solid particle transfer into the organic phase can be attributed to the design of the DTPS, including a height-adjustable second paddle that accounts for dynamic volume changes during the ongoing simulated GI transfer. Consequently, this prevents the second dissolution paddle from causing unwanted turbulences when stirring in the interface between the aqueous and organic phases upon increasing aqueous fluid volumes during the experiment. Finally, this enables gentle and controlled mixing of the two phases, allowing for a robust advanced dissolution test. Further, there was no observation of systematic issues, e.g., impaired drug partitioning via the aqueous:organic interface or mixing of the two phases potentially resulting in altered media characteristics. This is in line with a recent report published by O’Dwyer et al. with the conclusion that the mixing of an organic layer with FaSSIF components has only minimal effect [26]. In this study, the authors thoroughly studied the emulsification risk in a biphasic dissolution test setup and were able to demonstrate that neither the surface tension of FaSSIF nor the solubility of the model compounds ketoconazole and dipyridamole in FaSSIF, and their associated partitioning potential was affected by the decanol layer.

During the development and implementation of the DTPS, the impact of two important parameters on drug supersaturation/precipitation and partitioning were assessed, i.e., VR between aqueous and organic phase as well as the rate of first-order simulated GI transit (see also Section 2.3.4). Previously developed biphasic systems reported in the literature employ a variety of VRs, ranging from VR of 0.2 to 1.0, including small—and large—scale adaptations [12,13,16,17,20,27]. In the present study, two different VRs were evaluated (I) VR of 0.4 and (II) VR of 0.8 (see Section 2.3.4). The ratios were mainly selected due to technical considerations based on the USP II dissolution apparatus and linked to volumes often used for transfer/dissolution testing. Further, those ratios are well in line with previously reported ratios used by other research groups [12,13,16,17,20,27]. For discussion on the in vivo relevance of resulting area-to-volume ratios, the reader is referred to method Section 2.3.4. The present study shows that drug precipitation was significantly lower when employing a higher VR compared to the lower VR. In addition, the concentration of dissolved drug in the aqueous phase of the intestinal compartment was comparable in the case of both VRs tested. This shows that the higher amount of 1-decanol resulted in a higher rate and extent of the partitioning of MSC-A into the absorptive layer. Since the surface area available for partitioning of the drug between the two layers in the intestinal compartment was identical for both VRs, the apparent concentration of the drug in the 1-decanol layer appears to have a significant influence on the behavior of the drug in the aqueous intestinal phase as well. In fact, for the 75 mg dose level, the sink factor increase from 12.7 (for VR 0.4) to 20.3 (for VR 0.8) (equilibrium solubility in 1-decanol/apparent drug concentration in 1-decanol) may significantly contribute to increased mass transport. Therefore, the choice of VR can aid the modeling of the absorption of the drug, which is key when considering BCS class II and IV drugs.

A lot of progress has been made in the last couple of years, increasing the understanding of the role of gastric emptying on oral drug absorption. With the help of telemetric capsules, gastric residence times under fasted and fed state could be studied [28,29,30]. This enabled the integration of realistic gastric emptying kinetics and rates into in vitro tools as well as improving the awareness of variability as described by Grimm et al. [25]. Recent in vitro studies indicate that the rate of simulated gastric emptying can dictate the supersaturation potential and the degree of drug precipitation in simulated GI transfer experiments [6]. In the present study, partitioning into the absorptive sink medium as well as drug precipitation during experiments using 75 mg tablets were less impacted by the transfer rate, as compared to the different VRs, which can most likely be attributed to the high absorptive sink present in the in vitro assay. In the case of faster GI transit, a slightly faster partitioning was observed (Figure 4(D1)) since the total mass of API that is being transferred until a respective time point is much higher in the case of 5 min t_1/2_ compared to the experiment using 9 min t_1/2_. At the same time, not all the drug molecules partition instantly, which results in slightly higher initial precipitation using the faster transfer rate of 5 min t_1/2_ compared to the slower transfer rate of 9 min t_1/2_. Further, there was no marked difference in supersaturation, precipitation, and partitioning between different transfer rates at higher doses of 200 and 500 mg OS (see Supplementary Materials).

For an overall assessment of the DTPS, the developed biphasic in vitro tool was applied to predict the observations of an in vivo dose escalation study at a dose of 75, 200, and 500 mg. Due to the high permeability of MSC-A, the higher VR of 0.8 was chosen for further evaluation. In vitro data show a clear dose-dependency with higher doses resulting in higher amounts of dissolved and partitioned drugs. At 500 mg and 200 mg dose strength, the drug showed pronounced precipitation (comparing the total drug concentration to the aqueous phase concentration). However, an aqueous phase concentration plateau was reached for both doses indicating that an equilibrium between precipitation, re-dissolution, transfer, and partitioning was reached, maintaining the aqueous phase concentration at approximately 100 μg/mL for up to 40 min. 

**Figure 7:**
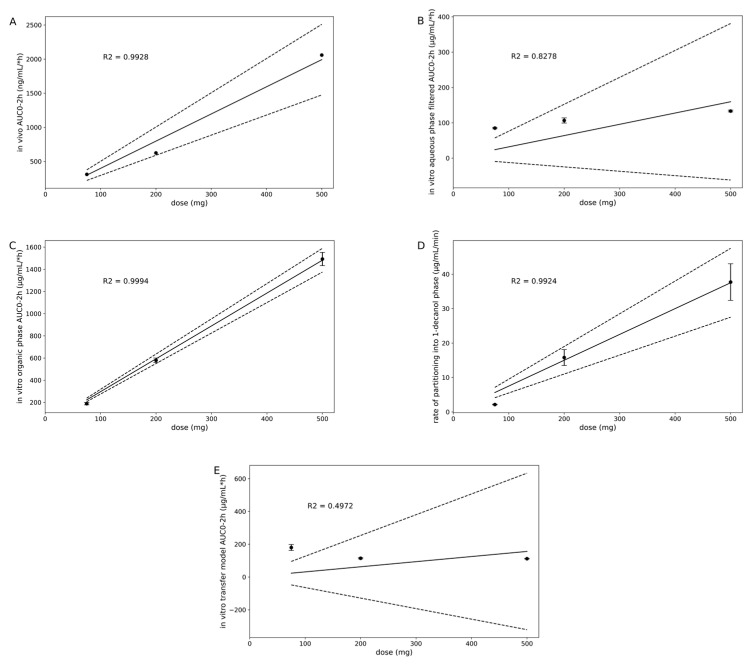
In vivo area under the plasma concentration-time curve of 75, 200, and 500 mg MSC-A as OS during first 2 h ((**A**)); slope = 3.9849; *p* = 0.0036). In vitro aqueous phase ((**B**); slope = 0.3191; *p* = 0.0902) and organic phase ((**C**); slope = 2.9639; *p* = 0.0003) area under the drug concentration-time curve during first 2 h, and rate of linear partitioning into organic phase between 7.5 and 20 min ((**D**); slope = 0.075; *p* = 0.0038) during in vitro DTPS experiments using 75, 200, and 500 mg MSC-A as OS. In vitro transfer model area under the drug concentration-time curve data obtained from experiments using 75, 200, and 500 mg MSC-A as OS ((**E**); slope = 0.3122; *p* = 0.2949). Dashed lines represent the upper and lower 95% confidence interval. For (**B**,**E**), data were fitted via a linear regression model for consistency purposes only. For aqueous phase data and associated saturated concentrations, the analysis results in poor R2 and *p* values of slope applying a linear model.

Drug concentration in the organic phase of the DTPS (from 75 mg to 200 mg to 500 mg) is in agreement with the observed AUC increase from a Ph1 clinical study (Table 3 and Figure 7A,C). The in vitro assessment of MSC-A revealed that the drug’s ability to supersaturate and the degree of supersaturation in dependence on the dose might be the key drivers for increased in vivo exposure. In addition, the maintenance of supersaturated concentrations for a prolonged period of time increased the amount partitioned into the organic phase and likely resulted in an increased exposure in vivo. Interestingly, the partitioning rate was observed to follow a linear relationship between 7.5 and 20 min for all of the three experiments, i.e., three dose levels. Further, the partitioning rate increased linearly with increasing doses, which can most likely be attributed to the increased mass transport in case of higher supersaturation values at higher dose levels (Figure 7D; *p* = 0.0038).

Using the in vitro transfer model setup, i.e., without an additional absorptive sink, supersaturation and precipitation were highly impacted by the dose used. Since all doses were administered as an OS, i.e., with no impact of a gastric dissolution step, the API concentration in the simulated gastric fluid as well as the simulated GI transfer rate, dictated the appearance of the API in the intestinal compartment. Thus, in vitro tmax was highest for the 500 mg dose and lowest for the 75 mg dose. Moreover, in vitro Cmax in the simulated intestinal compartment was lower for 75 mg compared to 200 mg and 500 mg, which is in agreement with the overall lower concentration in the simulated intestinal compartment for 75 mg compared to the higher doses. In vivo, gastric emptying under fasted state conditions is reported to be highly variable [25]. For example, the typical gastric emptying half-life reported in the literature ranges from 5 min to 15 min t_1/2_ [8,31,32,33,34]. The effect of different gastric emptying rates, i.e., 5 min vs. 9 min vs. 15 min t_1/2_, was studied previously and revealed no pronounced differences in drug supersaturation and precipitation of MSC-A [6]. As mentioned earlier, clinical plasma data revealed high dose linearity of MSC-A in healthy volunteers in Ph1 clinical trials (see Section 2.2). In contrast, dose linear drug dissolution in the simulated intestinal compartment was not observed using the in vitro transfer model approach, i.e., without an additional absorptive sink. Moreover, the AUC ratios between all doses tested, i.e., 200/75 mg, 500/200 mg, and 500/75 mg, were below one, indicating not only the absence of dose linear increase but, additionally, intense drug precipitation at higher dose levels (Table 4). In contrast, in vivo data suggest that MSC-A does not experience serious drug precipitation in the GI tract due to the dose-linear and nearly complete absorption within the first two hours after administration of 75–500 mg (Table 4). Furthermore, the observation of the overprediction of weakly basic drug precipitation in the semi-dynamic in vitro transfer model is in line with previous literature reports [8,35,36,37]. It is assumed that the lack of an in vitro sink, mimicking drug absorption, leads to unstable high drug supersaturation, which is questionable to appear in vivo to such a high degree as in an in vitro setup without an additional sink. Moreover, using the same aqueous volumes used in the DTPS experiments, i.e., 500 mL instead of 600 mL total aqueous volume, would have caused even more rapid drug precipitation due to the lower dose/volume ratio [6]. In the present study, MSC-A re-dissolution of precipitates could additionally not be observed due to still supersaturated concentrations until the end of the in vitro test for all three dose levels tested using the transfer model approach. In comparison to the standard transfer model, lacking an absorptive sink, the DTPS model indicated a reverse precipitation behavior. While MSC-A increasingly precipitated during the transfer in the standard transfer model, MSC-A precipitated amount (%) decreased towards the end of the transfer in the DTPS. This can be clearly attributed to the ongoing re-dissolution of precipitates driven by drug partitioning into 1-decanol and the associated shift in equilibrium in MSC-A solubility in the aqueous intestinal phase. Studying potential drug re-dissolution after initial precipitation can be extremely helpful in gaining a better understanding of the in vivo behavior of a drug substance or a drug in a specific formulation. Furthermore, this additional characteristic can increase the read-out of experiments aiming to support candidate selection, guide formulation development, and rank order different promising options during drug development.

## 5. Conclusions

Selecting the most suitable in vitro tool is essential to provide reliable predictions of the formulation performance in humans. Therefore, this study aimed to develop a more predictive in vitro model that combines the transfer from the stomach to the intestine as well as an absorptive sink in the intestinal compartment. The DTPS combines a biphasic in vitro dissolution system with a two-stage transfer model approach that captures physiologically relevant volumes as well as relevant gastric emptying kinetics. Further, an innovative 3D-printed paddle was designed, which was able to provide the desired hydrodynamics simultaneously in the simulated intestinal phase as well as in the organic layer. The advantage of this new dynamic paddle is the capability to adapt the paddle height of the organic layer paddle throughout the experiment when the intestinal phase increases in volume due to the continuous transfer of fluids from the simulated stomach compartment. Using the DTPS provided first valuable insights into the advanced assessment of supersaturation, precipitation, and re-dissolution behavior of MSC-A, a weakly basic development drug. In this study, the DTPS was successfully applied to estimate the dose linearity of MSC-A. Significant advantages were observed when comparing the results of the DTPS to a previously used USP II-based transfer model system. While precipitation for this drug is known not to impact its absorption significantly, standard pH-shift methods overestimate drug precipitation. In contrast, the DTPS accurately estimated the dose-linear supersaturation and partitioning. Overall, the DTPS provides the opportunity to obtain a more detailed picture of the precipitation behavior of weakly basic drugs and their (enabling) formulations. Given that both dissolution and absorption are key aspects driving the bioavailability of a drug, it is important to use reliable bio-predictive tools to account for both aspects to offer the advantage of streamlining the development process.

## Figures and Tables

**Table 1:** Physico-chemical characteristics and solubility in various media of MSC-A.

	MSC-A
MW	430 g/mol
Investigated human doses	75, 200, and 500 mg
Log P	~3
Basic pKa	5.8
Permeability	High
Solubility in SGFsp pH 2 (24 h)	3.5 ± 0.08 mg/mL [6]
Solubility in FaSSIF pH 6.5 (24 h)	0.027 ± 0.007 mg/mL [6]
Solubility in 1-decanol (24 h)	3.8 ± 0.01 mg/mL

MW, Molecular Weight; Solubility experiments have been conducted at 37 ± 0.5 °C, Log P and pKa determination at room temperature.

**Table 2:** Dosing conditions applied for FIH clinical trials.

Dose Per Dosing (mg)	Volume of OS (mL)	Additional Water (mL)
75	30	210
200	80	160
500	200	40

**Table 3:** 3D printing settings.

Parameter	Setting
Nozzle	0.4 mm
Material	PLA
Temperature nozzle	215 °C
Temperature print bed	60 °C
Perimeter print speed	45 mm/s
Layer height	0.15 mm
Perimeter	2
Solid layers	7
Infill	2%

**Table 4:** Comparison of area under the plasma concentration-time curve vs. area under the concentration-time curve obtained from in vitro transfer model experiments vs. area under the concentration-time curve of the aqueous dissolved amount and the organic partitioned amount obtained from in vitro DTPS experiments. Clinical data represent means (*n* = 12 for 75 mg, and *n* = 6 for 200 and 500 mg).

AUC_0–120min_ (µg/mL × h)	OS 75 mg	OS 200 mg	OS 500 mg	OS 200 mg/ OS 75 mg	OS 500 mg/ OS 200 mg	OS 500 mg/ OS 75 mg
Transfer model data	179.8 ± 18.1	114.4 ± 3.5	111.8 ± 1.5	0.63	0.98	0.62
DTPS data aqueous	85.1 ± 2.1	106.6 ± 7.4	133.3 ± 2.6	1.25	1.25	1.57
DTPS data organic	188.1 ± 4.4	579.21 ± 23.0	1492.5 ± 76.3	2.46	2.58	7.93
Plasma data	0.31	0.625	2.059	2.02	3.29	6.64

## Data Availability

Not applicable.

## Supplementary Materials

The following supporting information can be downloaded at https://www.mdpi.com/article/10.3390/pharmaceutics15041069/s1. Table S1. HPLC - analytical conditions; Table S2. Gradient profile of solubility-indicating HPLC method for MSC-A; Table S3. UPLC-MS/MS method; Figure S1. Aqueous phase drug concentration profiles (A), total amount profiles (aqueous phase & precipitate) (B), and precipitated amount (C) obtained from the aqueous filtered and un-filtered phase; partitioning profiles (D1 concentration/time vs. D2 mass/time) obtained from the organic phase during in vitro DTPS experiments using VR of 0.8 at simulated gastric emptying rates of 5 min half-time vs. 9 min half-time and 200 mg MSC-A as oral solution. Means ± SD, n = 3; Figure S2. Aqueous phase drug concentration profiles (A), total amount profiles (aqueous phase & precipitate) (B), and precipitated amount (C) obtained from the aqueous filtered and un-filtered phase; partitioning profiles (D1 concentration/time vs. D2 mass/time) obtained from the organic phase during in vitro DTPS experiments using VR of 0.8 at simulated gastric emptying rates of 5 min half-time vs. 9 min half-time and 500 mg MSC-A as oral solution. Means ± SD, n = 3.
