# Supplementary material for: Development and Application of a Dissolution-Transfer-Partitioning System (DTPS) for Biopharmaceutical Drug Characterization
Source: Pharmaceutics. 2023 Mar 26;15(4):1069. doi: 10.3390/pharmaceutics15041069 (PMC10145677; doi:10.3390/pharmaceutics15041069)
Supplement: Supplementary file 1 [file pharmaceutics-15-01069-s001.zip › pharmaceutics-2278554-supplementary.pdf]

Supplementary data

Table S1. HPLC - analytical conditions

	**MSC-A**
Column	Phenomenex Kinetex 2.6 µm XB-C18 100 A (50 x 4.6 mm)
Flow rate (mL/min)	1.4
Column temperature (°C)	40
Wavelength detection (nm)	227
Injection volume (µL)	90
Retention time (min)	2.1

Table S2. Gradient profile of solubility-indicating HPLC method for MSC-A.

**Time (min)**	**Solvent A (%)***	**Solvent B (%)****
0	0	100
3.5	0	100
3.6	85	15
5.5	85	15

*Solvent A: 5% acetonitrile, 95% water mixed with 0.1% (V/V) TFA

**Solvent B: 95% acetonitrile, 5% water mixed with 0.1% (V/V) TFA

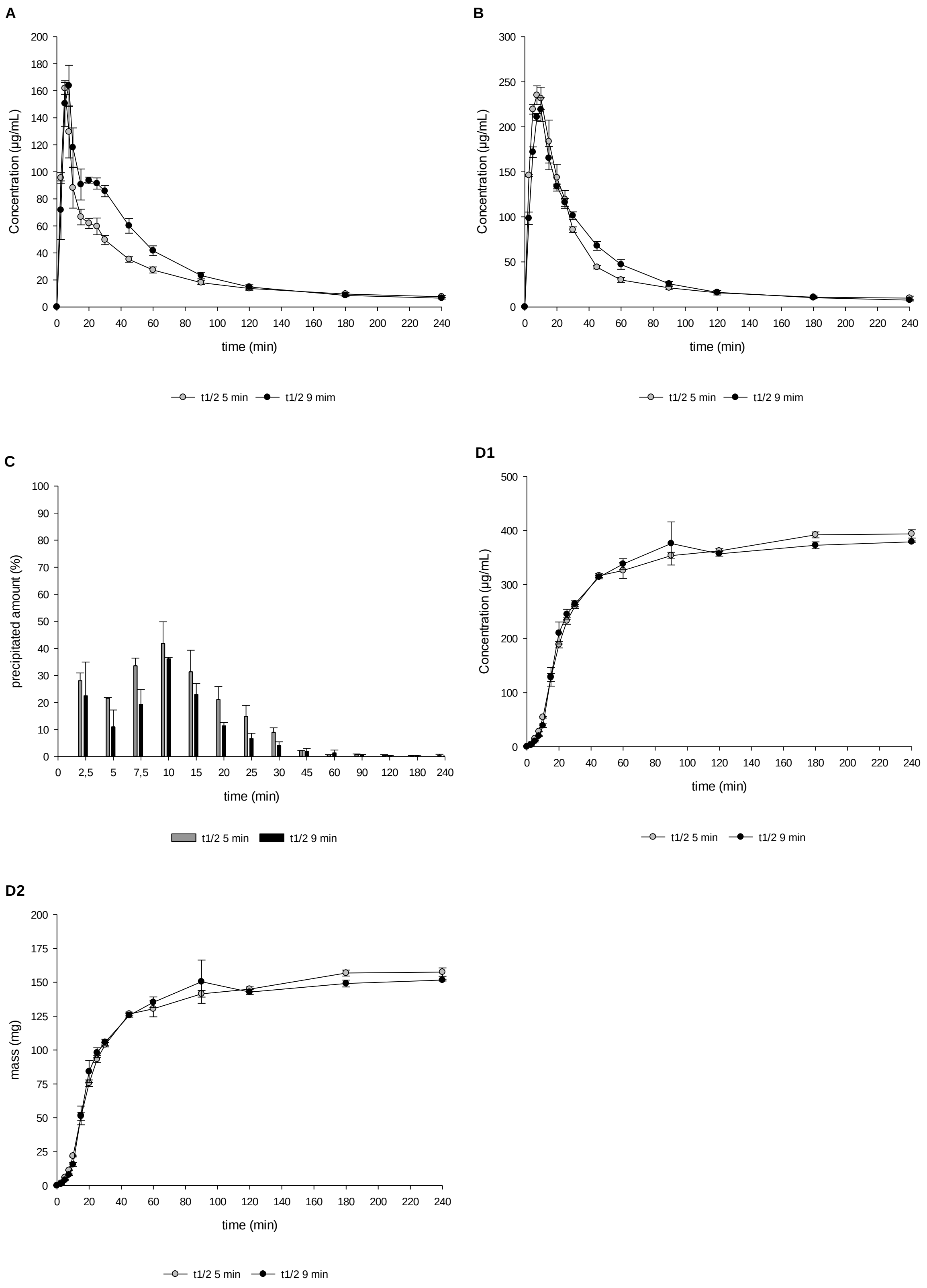

Figure S1. Aqueous phase drug concentration profiles (A), total amount profiles (aqueous phase & precipitate) (B), and precipitated amount (C) obtained from the aqueous filtered and un-filtered phase; partitioning profiles (D1 concentration/time *vs*. D2 mass/time) obtained from the organic phase during *in vitro* DTPS experiments using VR of 0.8 at simulated gastric emptying rates of 5 min half-time *vs*. 9 min half-time and 200 mg MSC-A as oral solution. Means ± SD, n = 3.

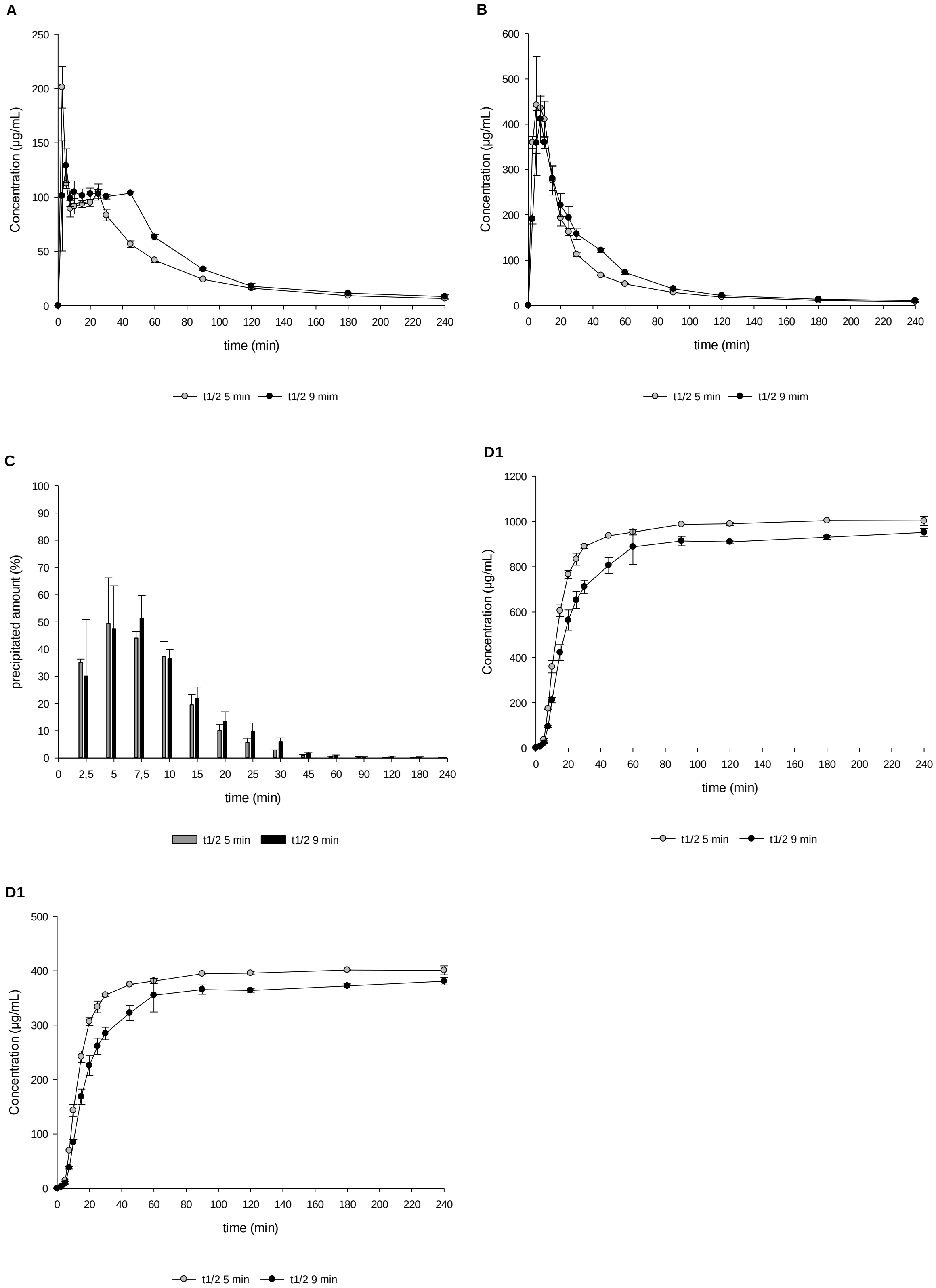

Figure S2. Aqueous phase drug concentration profiles (A), total amount profiles (aqueous phase & precipitate) (B), and precipitated amount (C) obtained from the aqueous filtered and un-filtered phase; partitioning profiles (D1 concentration/time *vs*. D2 mass/time) obtained from the organic phase during *in vitro* DTPS experiments using VR of 0.8 at simulated gastric emptying rates of 5 min half-time *vs*. 9 min half-time and 500 mg MSC-A as oral solution. Means ± SD, n = 3.

Table S3. UPLC-MS/MS method

<table>
<tr><td>LC System:</td><td colspan="4">Waters Acquity UPLC® I-Class System
(Binary Solvent Manager with Acquity UPLC® Sample Manager-FL equipped with Masslynx version 4.1)</td></tr>
<tr><td>Column:</td><td colspan="4">Waters Acquity UPLC BEH C_{18}, 1.7 µm, 2.1 x 50 mm</td></tr>
<tr><td>Sample temperature:</td><td colspan="4">4 °C</td></tr>
<tr><td>Column temperature:</td><td colspan="4">40 °C</td></tr>
<tr><td>Mobile Phase A:</td><td colspan="4">1000:10 water/1 M ammonium formate</td></tr>
<tr><td>Mobile Phase B:</td><td colspan="4">acetonitrile</td></tr>
<tr><td rowspan="9">Gradient Program:</td><td rowspan="2">Time (min)</td><td>Mobile Phase A</td><td>Mobile Phase B</td><td rowspan="2">Curve</td></tr>
<tr><td colspan="2">800 µL/min total flow rate</td></tr>
<tr><td>0.00</td><td>65%</td><td>35%</td><td>Initial</td></tr>
<tr><td>1.50</td><td>65%</td><td>35%</td><td>6</td></tr>
<tr><td>1.60</td><td>5%</td><td>95%</td><td>6</td></tr>
<tr><td>2.60</td><td>5%</td><td>95%</td><td>6</td></tr>
<tr><td>2.70</td><td>65%</td><td>35%</td><td>6</td></tr>
<tr><td>3.00</td><td>65%</td><td>35%</td><td>6</td></tr>
<tr><td>3.20</td><td>Stop</td><td>Stop</td><td>NA</td></tr>
<tr><td>Seal Wash</td><td colspan="4">50:50 methanol/water (5 min)</td></tr>
<tr><td>Strong wash solvent:</td><td colspan="4">1000:5 acetonitrile/formic acid</td></tr>
<tr><td>Strong wash volume:</td><td colspan="4">1500 µL</td></tr>
<tr><td>Weak wash solvent:</td><td colspan="4">10:90 acetonitrile/water</td></tr>
<tr><td>Weak wash volume:</td><td colspan="4">2500 µL</td></tr>
<tr><td>Injection volume (standard):</td><td colspan="4">10 µL</td></tr>
<tr><td>Injection mode:</td><td colspan="4">Partial loop</td></tr>
<tr><td>Injection loop:</td><td colspan="4">20 µL</td></tr>
<tr><td>Injection needle:</td><td colspan="4">10 µL</td></tr>
<tr><td>Cycle injection valve</td><td colspan="4">2.4 min, 2.9 min</td></tr>
<tr><td>Method notes:</td><td colspan="4">Use 0.0025” i.d. tubing between LC column and MS source port to improve separation.</td></tr>
<tr><td>Detector:</td><td colspan="4">Triple Quadrupole MS-Detector AB SCIEX API 4000</td></tr>
<tr><td>Software:</td><td colspan="4">AB SCIEX Analyst, version 1.4.2</td></tr>
<tr><td>Ion source</td><td colspan="4">Turbo Ion Spray</td></tr>
<tr><td>Scan Type</td><td colspan="4">Selected reaction monitoring (SRM) (MS/MS)</td></tr>
<tr><td>Polarity:</td><td colspan="4">Positive</td></tr>
<tr><td>Acquisition time</td><td colspan="4">2.0 min</td></tr>
<tr><td>Pause time</td><td colspan="4">25 ms</td></tr>
<tr><td>Minimum total cycle time</td><td colspan="4">Approximately 4 min</td></tr>
<tr><td>Q1 Resolution</td><td colspan="4">Unit</td></tr>
<tr><td>Q3 Resolution</td><td colspan="4">Unit</td></tr>
</table>

Retention time MSC-A: approximately 1.05 min (± 0.21 min)

Mass range MSC-A: 430 → 279 @100 ms
